# Supplementary material for: Bacterial Nanocellulose Fortified with Antimicrobial and Anti-Inflammatory Natural Products from Chelidonium majus Plant Cell Cultures
Source: Materials (Basel). 2021 Dec 21;15(1):16. doi: 10.3390/ma15010016 (PMC8746069; doi:10.3390/ma15010016)
Supplement: Supplementary file 1 [file materials-15-00016-s001.zip › materials-1496608-supplementary.pdf]

Supplementary Material for the article

Bacterial nanocellulose fortified with antimicrobial and anti-inflammatory natural products from *Chelidonium majus* plant cell cultures

Sylwia Zielińska[1], Adam Matkowski[2*], Karolina Dydak[3], Monika Ewa Czerwińska[4,5], Magdalena Dziągwa-Becker[6], Mariusz Kucharski[6], Magdalena Wójciak[7], Ireneusz Sowa[7], Stanisława Plińska[8], Karol Fijałkowski[9], Daria Ciecholewska-Juśko[9], Michał Broda[9,10], Damian Gorczyca[11], Adam Junka[3]

[1]Department of Pharmaceutical Biology and Biotechnology, Division of Pharmaceutical Biotechnology, Wroclaw Medical University, 50-556 Wrocław, Poland
[2]Department of Pharmaceutical Biology and Biotechnology, Division of Pharmaceutical Biology and Botany, Wroclaw Medical University, 50-556 Wroclaw, Poland
[3]Pharmaceutical Microbiology and Parasitology, Wroclaw Medical University, 50-556 Wroclaw, Poland
[4]Department of Biochemistry and Pharmacogenomics, Faculty of Pharmacy, Medical University of Warsaw, 02-097 Warszawa, Poland
[5]Centre for Preclinical Research, Medical University of Warsaw, 1B Banacha street, 02-097 Warsaw, Poland
[6]Department of Weed Science and Tillage Systems, Institute of Soil Science and Plant Cultivation State Research Institute, 50-540 Wrocław, Poland
[7]Department of Analytical Chemistry, Medical University of Lublin, 20-093 Lublin, Poland
[8]Department of Inorganic Chemistry, Wroclaw Medical University, 50-556 Wrocław, Poland
[9] Department of Microbiology and Biotechnology, Faculty of Biotechnology and Animal Husbandry, West Pomeranian University of Technology, 70-311 Szczecin, Poland
[10] Pomeranian-Masurian Potato Breeding Company, 76-024 Strzekęcino, Poland
[11] Faculty of Medicine, Lazarski University, 02-662 Warszawa, Poland
*Correspondence: pharmaceutical.biology@wp.eu; Tel.: +48717840497

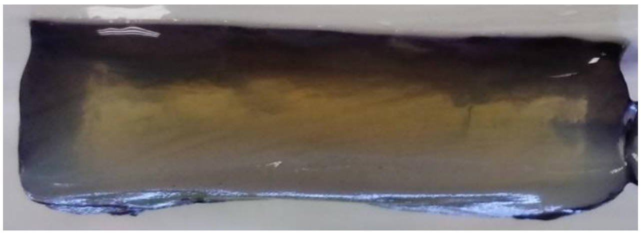

Bottom of BNC carrier

Figure S1. Vertical cross-section BNC containing *C. majus* cells. The blue areas show regions containing cells – mainly on the top of BNC carrier and on the side parts of BNC carrier. The middle and bottom parts are dye-free, suggesting lack of live *C. majus* cells in these areas.

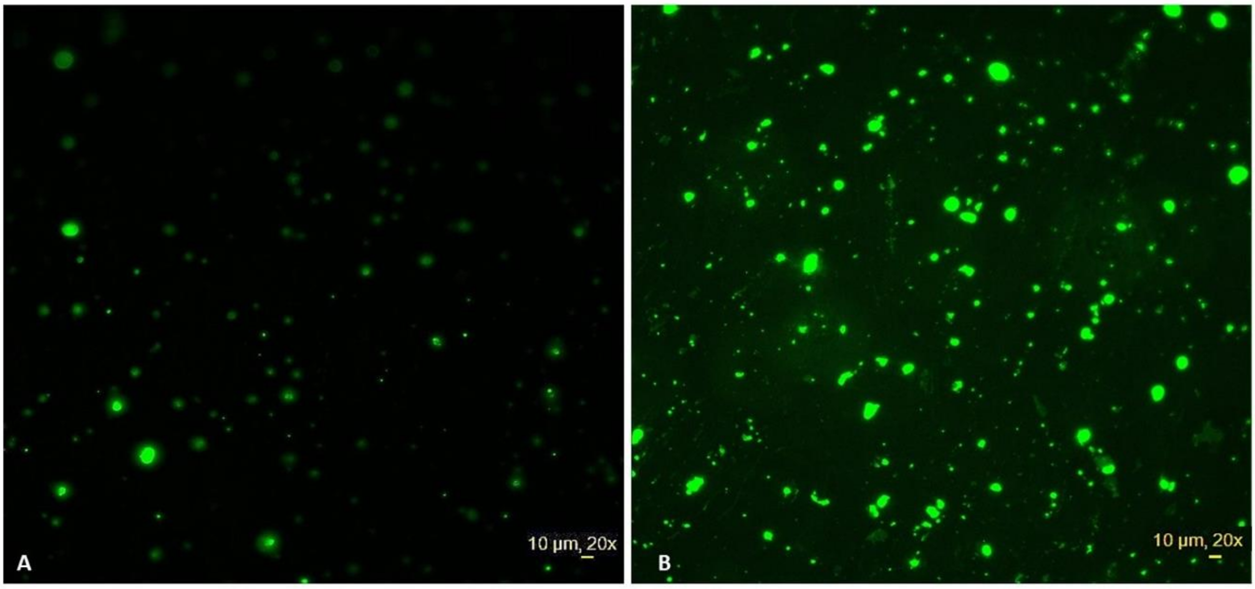

Figure S2. *C. majus* **cells viability measured after two (A) and four (B) weeks of culture on liquid medium MH3. The yellow bar shows length of 10µm, magnification 20×, fluorescent microscope Lumascope 620.**

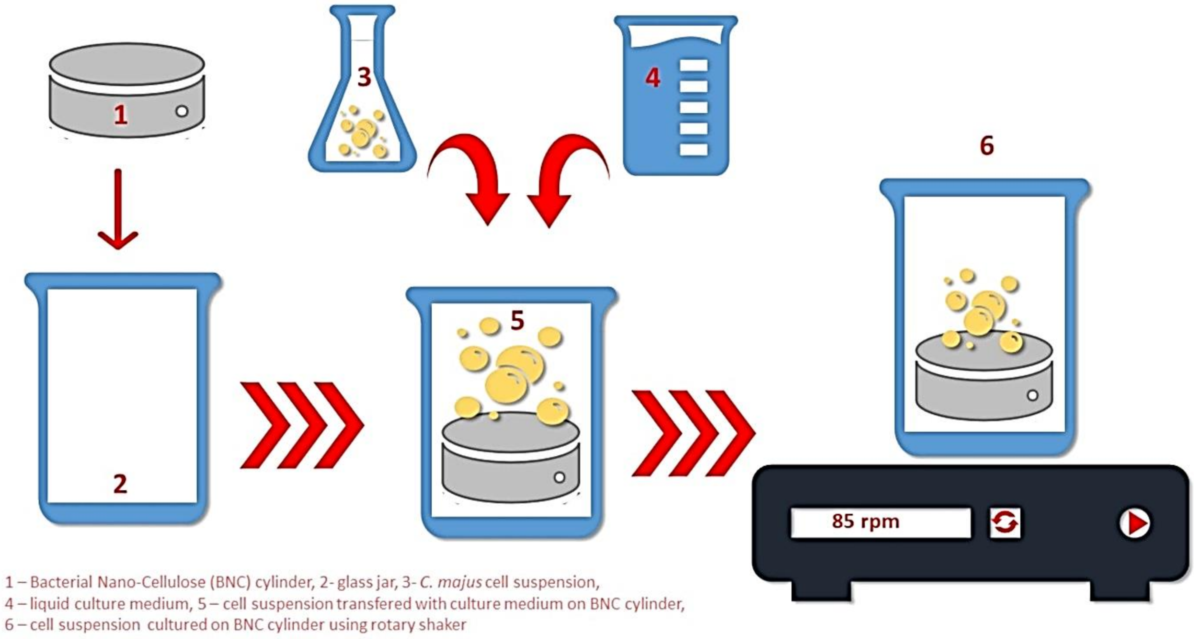

Figure S3. The schematic processes of the ***C. majus*** **cells cultured on Bacterial Nano-Cellulose (BNC) matrix.**

Table S1. Minimal Inibitory Concentration and Minimal Biofilm Eradication Concentration of liquid obtained from BNC matrices

type of BNC	Minimal Inhibitory Concentration [% v/v]			Minimal Biofilm Eradication Concentration [% v/v]		
	S. aureus	*P. aeruginosa*	*C. albicans*	*S. aureus*	*P. aeruginosa*	*C. albicans*
3d BC- NC	25%	50%	above tested range of concentrations	above tested range of concentrations	above tested range of concentrations	above tested range of concentrations
3d BC - H_2O	above tested range of concentrations	above tested range of concentrations	above tested range of concentrations	above tested range of concentrations	above tested range of concentrations	above tested range of concentrations
3d BC-NaOH	above tested range of concentrations	above tested range of concentrations	above tested range of concentrations	above tested range of concentrations	above tested range of concentrations	above tested range of concentrations

Table S2. The impact of MH3 medium on tested microbes viability. The viability is presented as [%] in comparison to standard microbiological Tryptic Soy Broth (for *S. aureus*, *P. aeruginosa*, *C. albicans*) or Herstin-Schramm (for *K.xylinus*) liquid medium within 1 day, 3 days and 7 days of incubation.

species	1 day	3 days	7 days
S. aureus	65.00±11.63	20.00±7.40	5.25±3.31
P. aeruginosa	61.75±11.33	36.25±6.44	10.50±6.76
C. albicans	64.75±15.01	70.50±11.36	23.00±6.06
K. xylinus	43.00±9.30	28.50±6.60	6.50±2.01

Table S3. LC-MS/MS analysis of the content of the detected compounds and their retention times (Rt) in 3-, 5-, and 7-day-old cellulose (NaOH, H_2O, NC) with *C. majus* cells cultured on MH3 medium for 14, 21, and 28 days.

Compound	Rt [min]	NC [µg/ml ± SE]									H_20 [µg/ml ± SE]			NaOH [µg/ml ± SE]		
		3*	3**	3***	5*	5**	5***	7*	7**	7***	3*	5*	7*	3*	5*	7*
malic acid	1.08	p	p	p	p	p	p	p	p	p	p	p	nd	p	p	nd
trans-aconitic acid	1.15	p	nd	nd	p	p	p	p	p	p	p	p	nd	p	p	nd
quinic acid	1.30	nd	p	p	p	p	p	p	p	p	p	nd	nd	p	nd	nd
protocatechuic acid	1.54	nd	p	p	nd	p	p	p	p	p	nd	nd	nd	nd	nd	nd
chlorogenic acid	2.51	nd	11.65 ±0.52	12.166 ±1.314	3.875 ±0.325	3.77 ±0.33	4.225 ±0.369	LOQ	LOQ	LOQ	7.800 ±0.400	LOQ	nd	LOQ	nd	nd
trans-caffeic acid	2.71	nd	1.400 ±0.144	1.550 ±0.10	0.250 ±0.000	0.220 ±0.017	LOQ	LOQ	LOQ	LOQ	0.411 ±0.031	LOQ	nd	LOQ	nd	nd
salicylic acid	2.79	nd	0.058 ±0.008	0.075 ±0.000	0.050 ±0.000	nd	0.110 ±0.026	LOQ	LOQ	LOQ	LOQ	nd	nd	nd	nd	nd
tannic acid	2.84	nd	1.000 ±0.100	nd	nd	nd	2.833 ±0.333	LOQ	LOQ	LOQ	LOQ	nd	nd	nd	nd	nd
a hydroxybenzoic acid	2.85	nd	0.050 ±0.000	0.075 ±0.00	0.005 ±0.000	0.075 ±0.00	0.016 ±0.001	LOQ	LOQ	LOQ	LOQ	nd	nd	nd	nd	nd
p-coumaric acid	3.97	nd	nd	0.150 ±0.027	nd	nd	LOQ	LOQ	LOQ	LOQ	LOQ	nd	nd	nd	nd	nd
vanillin	5.15	nd	nd	0.350 ±0.028	nd	nd	LOQ	LOQ	LOQ	LOQ	LOQ	nd	nd	nd	nd	nd
protopine derivative	6.75	p	p	p	p	p	p	p	p	p	p	p	nd	p	p	nd
coptisine	7.50	LOD	nd	nd	nd	nd	nd	nd	nd	nd	LOQ	0.239 ±0.001	LOQ	LOQ	LOQ	LOQ
quercetin	8.34	LOD	1.050 ±0.020	0.966 ±0.016	0.500 ±0.086	0.450 ±0.028	0.490 ±0.036	LOQ	LOQ	LOQ	LOQ	0.090 ±0.00	nd	nd	nd	nd
chelidonine	9.56	LOD	LOQ	LOQ	LOQ	LOQ	LOQ	nd	nd	nd	LOD	nd	0.064 ±0.088	nd	nd	0.081 ±0.004
chelerythrine	10.44	nd	nd	nd	nd	nd	LOD	nd	nd	0.062 ±0.034	0.125 ±0.029	0.074 ±0.001	nd	nd	nd	nd
sanguinarine	11.80	LOQ	0.050 ±0.000	LOD	0.050 ±0.000	0.030 ±0.008	0.011 ±0.001	nd	0.034 ±0.020	0.152 ±0.014	0.022 ±0.002	0.100 ±0.007	0.576 ±0.060	nd	nd	0.643 ±0.075
protopine	12.76	6.250 ±0.750	8.500 ±0.764	5.833 ±0.601	4.750 ±0.433	3.750 ±0.144	5.940 ±0.314	LOD	LOQ	LOQ	7.566 ±0.929	LOQ	0.197 ±0.019	LOQ	LOQ	0.395 ±0.012

p – present, where identification was based on mass spectra with no reference substances; nd – not detected; * after 14 days, ** after 21 days and *** after 28 days

Table S4. Isoquinoline alkaloids content [µg/ml ± SD] in the extracts of *C. majus* callus cultured for 4 weeks on solid medium MH3 supplemented or not with plant growth regulators (PGRs: 2,4-Dichlorophenoxyacetic acid [2.5 µM] 1-Naphthaleneacetic acid [0.5 µM] Kinetin [1.0 µM])*, and in liquid MH3 medium supplemented or not with the same composition of PGRs after the 4 week of *C. majus* cell suspension culture.**

compound	Callus MH3*	Callus MH3+PGRs*	Cell-free culture medium MH3**	Cell-free culture medium MH3+PGRs**
Protopine	0.2950±0.0126	0.5886±0.0103	0.3005±0.0058	0.2934±0.0155
Chelidonine	0.0567±0.0042	0.1802±0.0032	0.0441±0.0082	0.0595±0.0042
Coptisine	LOQ	0.1667±0.0058	LOQ	0.1471±0.0172
Sanguinarine	0.1617±0.0051	0.6670±0.0652	0.2628±0.0214	0.5389±0.0490
Chelerythrine	nd	0.1370±0.0030	nd	nd

**C. majus* callus extracts were prepared and analyzed according to the previously reported method by Zielińska et al. 2018a [8]; **culture media after *C. majus* cells separation using filter paper.

Table S5. Antimicrobial activity of free *C.majus* metabolites-enriched MH3 medium

	Minimal Microbiocidal Concentration [%v/v]			Minimal Biofilm Eradication Concentration [% v/v]		
microbes	*S. aureus*	*P. aeruginosa*	*C. albicans*	*S. aureus*	*P. aeruginosa*	*C. albicans*
free *C.majus* metabolites-enriched medium	50%	25%	50%	above tested range of concentration	above tested range of concentration	above tested range of concentration
